# Personal healthcare

**DOI:** 10.1051/ject/2025020

**Published:** 2025-06-16

**Authors:** Raymond K Wong

As we age, we encounter more medical ailments; there is nothing profound in that statement. But it becomes more significant when it affects you personally. Yes! I was on the other side of the healthcare system this past spring. And like many healthcare colleagues have affirmed, undergoing the knife ourselves gives one a new appreciation for what our patients experience. I know of at least three other clinician colleagues, JECT editorial board members, and/or our peer reviewers who also had serious medical episodes in recent months. Thankfully, all four of us are safely on the other side and mending well. Fortunately for me, I did not undergo cardiopulmonary bypass procedures, but two of us did. Still, I was intubated three times and had a Foley catheter once (none of which were fun experiences), and I have a pretty lengthy scar down my neck.

Overall, I had great medical care from an elite medical institution, but even there, complications can arise. Through personal experience, I am reminded that little mistakes can lead to significant consequences. A minor nick became a serious infection, for example. While we perfusionists are not at risk of causing minor nicks on patients, there are plenty of other minor things that we do, or neglect to do, that can become more significant for our patients. Simple precautions to avoid gaseous microemboli can help our patients’ neurological recovery as they wake up from the haze of anesthesia, or taking action to increase our oxygen delivery might make the difference in whether or not our patients void their bladders on their own or require renal replacement therapy. We perfusionists are privileged to provide care for our patients, and it is important to remind ourselves how consequential our actions or inactions are, even when they seem trivial.

On a personal level, I would like to thank everyone who pitched in to keep JECT functions moving along during the time I was out on medical leave, including folks at AmSECT, our publishers, EDP Sciences, as well as our associate editors. Thanks also to our authors for being patient with any delays they might have encountered in the peer review process as we work through a little backlog.

Googling the title of this editorial will lead you to lots of links for personalized healthcare providers. But my intention is to refer to the self-care of us clinicians. My purpose here is to remind JECT readers to take care of their own personal healthcare needs despite often-hectic clinical duties. I had put off being diagnosed for more than a year but thankfully that has not seemed to affect my outcomes. My delay could just as easily have turned out to be much more consequential. Keeping ourselves healthy only allows us to serve our patients better.

